# The role of quality control circles in sustained improvement of medical quality

**DOI:** 10.1186/2193-1801-2-141

**Published:** 2013-04-02

**Authors:** Lin-run Wang, Yang Wang, Yan Lou, Ying Li, Xing-guo Zhang

**Affiliations:** Department of Pharmacy, The First Affiliated hospital, College of Medicine, Zhejiang University, 79 QingChun Road, Hangzhou, Zhejiang, 310000 People’s Republic of China

**Keywords:** Quality control circle (QCC), Medical quality

## Abstract

We used quality control circles (QCC) followed by the PDCA Deming cycle and analyzed the application of QCC to the sustained improvement of a medical institution in Zhejiang province. Analyses of the tangible and intangible achievements of QCC revealed that the achievement indices for reductions in internal errors, reductions in costs, improvements in the degree of patient satisfaction, improvements in work quality, and improvements in economic performance were 109.84% ± 16.47%, 135.04% ± 50.33%, 126.26% ± 53.69%, 100.58% ± 22.83%, and 104.07% ± 5.45%, respectively. The improvements in these areas were 61.12% ± 13.2%, 60.47% ± 28.91%, 34.41% ± 22.96%, 49.22% ± 25.39%, and 73.70% ± 5.24%, respectively. The intangible achievements were reflected as follows: 5% of QCC members showed an activity growth value of 1–2 points, 83% 1–2 points, 12% more than 2 points. As a result, QCC activity showed prominent results in fostering long-lasting improvement in the quality of medical institutions in terms of both tangible and intangible factors. In short, QCC can be used as an effective tool to improve medical quality.

## Introduction

The content and goals of modern QCC have outgrown their original range in business management. QCC has been gradually applied in the domestic medical and healthcare fields. Its goal is to increase the morale of medical workers by improving their awareness of spotting and solving medical problems, improving medical working environments, and eventually increasing the quality of medical care, reducing the costs of medical management, and increasing the efficiency of medical services.

During the past 30 years of China’s reform and opening up, the medical quality management system has been basically established. On one hand, the administrative department of health has begun to give full attention to the medical continuous quality improvement and medical safety management, and formed a good medical quality management philosophy. On the other hand, with the development of the market economy system, the hospitals formed the quality consciousness to some extent, and gradually pay attention to the construction of medical quality.

Quality circles were first established in Japan in 1962; Kaoru Ishikawa has been credited with their creation. The movement in Japan was coordinated by the Japanese Union of Scientists and Engineers (JUSE). The first circles were established at the Nippon Wireless and Telegraph Company but then spread to more than 35 other companies in the first year. By 1978 it was claimed that there were more than one million quality circles involving some 10 million Japanese workers. They are now in most East Asian countries; it was recently claimed that there were more than 20 million quality circles in China Currently, QCC has been widely established in hospitals in Taiwan ( Chang et al. 2010 ; Lin et al. 2009 ), Japan ( Helmer & And Gunatilake 1988 ), Germany ( Wensing et al. 2009 ; Wensing et al. 2004 ), and Australia ( Spiegel et al. 2012 ) and has achieved prominent results.

QCC has been gradually introduced to medical institutions in China since 2001. Activities included reducing the number of internal errors in outpatient prescription dispensation, improving the work quality of operating room nursing, reducing the rate of complications caused by venous indwelling needles, and increasing the inspection rate of urine and stool samples of orthopedics patients. These achievements have involved the areas of nursing technique and management ( Zhong et al. 2002 ), pharmacy management ( Wang et al. 2010 ; Zhang et al. 2009 ; Zhang et al. 2010 ), operating room management ( Wang 2009 ), and other types of quality management ( Tong 2009 ). The results were pronounced, and the quality and efficiency of corresponding departments were noticeably improved.

Since 2008, there were successive QCC activities and activity symposiums initiated by hospitals in Shanghai, Zhejiang, Hainan, Jiangsu, and Jiangxi. The provincial Hainan government used the opportunity to start third-party evaluations of hospitals, including QCC as part of quality control. QCC activities have been widely promoted throughout Hainan.

The QCC activities carried out in Zhejiang met the era’s requirement for sustained improvement of medical quality. Under the direction of the administrative department of healthcare, a QCC activity promotion work team was established in the hospital pharmacy management quality control center. With the combined efforts of the Zhejiang Medical Association, Zhejiang Hospital Association, Zhejiang Hospital Pharmacy Management Quality Control Center, and Zhejiang Nursing Association, the first and second QCC activity achievement symposiums and the third provincial hospital QCC activity initiation meeting were successfully held. Until now, there have been over 200 circle activities. In the provincial rank hospital appraisal, QCC activity has become a hard criterion for assessing the performance of hospital management. Reductions in internal errors, reductions in costs, improvements in the degree of satisfaction, improvements in work quality, and improvements in economic performance are the main theme activities that QCC carries out. These have greatly promoted hospitals’ active roles in quality management and control. They have also promoted the application and practice of advanced tools for quality control in our country.

## Materials and methods

### Formation of QCC and the execution of QCC activity

Training: The purpose, operational procedure, and methods by which QCC activities were to be carried out were taught by trainers, who placed a focus on strengthening ideas and reaching consensus in order to encourage trainees establish a “hoping to do, wanting to do and able to do” attitude. The training took place through combinations of class training and individual training. All employees were trained in classes; and heads and counselors were trained individually with mock practice. A combination of theoretical teaching before execution and practical guidance during execution was adopted.

Forming the circle: The objective of QCC is to solve problems by relying on the personnel of the same department (workplace). To condense the QQC centripetal force, guarantee smooth execution of QCC activities, and achieve QCC goals, participating members form a circle and announce with determination and will that they will improve quality. Effective circling formation typically starts with finding the right candidates. The optimal number of QCC members should be about 5–10, and the members should come primarily from the same department or workplace. Each participating QCC member should have a corresponding work responsibility. Running QCC in hospitals is the responsibility of all members, but different members should take charge in different specific areas. Generally speaking, duty assignments can be based on whether the member is directly or indirectly related. Typical roles include project director, liaison, counselor, circle head, and circle member.

Execution: QCC activity starts with the circle head and members using *in situ* data and materials to look for actual problems through brainstorming. Ideas are then analyzed and modified with quality management approaches. In QCC activities, circle meetings are the most important form. Good circle meetings promote the smooth execution of the whole QCC process and provide solid tangible and intangible achievements. Bad circle meetings, i.e., meetings without discussion, discussion without decision, decision without execution, hinder QCC activity. The 10 steps of QCC activity are theme selection, activity planning, understanding status quo, setting goals, analysis, formulating strategy, execution and evaluation, confirmation of results, standardization, review, and improvement. The circle meeting should be held monthly and an activity log should be maintained. One cycle of QCC activity lasts 6 months.

### Sources of materials

The data were based on the first and second phases and parts of the third phase of the QCC activity that took place in a Zhejiang medical institution between Jan 2009 and June 2011. Materials from the 92 QCCs uniformly trained and executed under the supervision of hospital pharmacy management quality control center, mainly including activities carried out by pharmaceutical and nursing departments, were summarized and analyzed.

### Summarization and organization of the data

**Figure 1:**
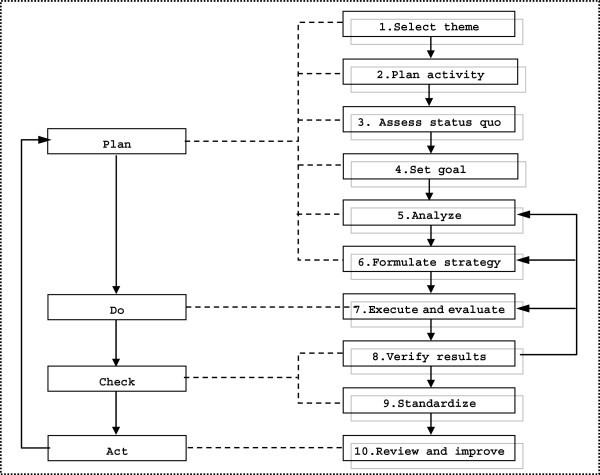
**Basic steps of QCC activities.**

Based on the characteristics of the QCC theme activities, we summarized the data in the following respects: reductions in internal errors, reductions in costs, improvements to the degree of patient satisfaction, improvements in work quality, and improvements in economic performance (Table 1). The basic steps of QCC activity generally followed the Deming cycle (PDCA cycle). The four stages, plan, do, check, and act, were realized through 10 basic steps (Figure 1). The performance of QCC activity was evaluated in terms of tangible and intangible achievements.

**Table 1:** **Basic data of QCC activities (n = 92)**

Category index	Number of cases	Percentage (%)
Department		
Pharmacy	54	58.7
Nursing	37	40.2
Drug company branch	1	1.1
Theme activities		
Reductions in internal error	55	59.8
Reductions in costs	4	4.3
Improvements in degree of satisfaction	14	15.2
Improvements in work quality	16	17.4
Improvements in economic performance	3	3.3

### Statistical analysis of the data

The evaluation indices for tangible achievements included the following: Achievement Index = [(data value after improvement – data value before improvement)/(goal value - data value before improvement)] × 100%; Improvement Index = [(data value after improvement – data value before improvement)/data value before improvement] × 100%. For intangible achievements, evaluations were made regarding such aspects as the ability to solve problems, sense of responsibility, communication and coordination, team cohesiveness, self-confidence, and approach to quality control. As previous described ( Wensing et al. 2009 ; Spiegel et al. 2012 ), each aspect was marked by self-evaluation of the QCC members, with the highest possible score being 5 points and the lowest 1. The Activity Growth Value = mean mark after activity – mean mark before activity. Activity Growth Value was used to assess the intangible achievement: positive values indicated improvement and negative values indicated deterioration. All data were represented as ± SD.

## Results

The results of QCC activity were mainly reflected in tangible and intangible achievements.

### Tangible achievements

For tangible achievements, data were recorded based on the QCC theme activity such as reductions in internal errors, reductions in costs, improvements in the degree of patient satisfaction, improvements in work quality of work, and improvements in economic performance. Scores were recorded (before improvement vs. after improvement) in the following way:

As shown in Table 2, after each goal value was set, the achievement indices were 109.84% ± 16.47% for reductions in internal errors, 135.04% ± 50.33% for reductions in costs, 126.26% ± 53.69% for improvements in the degree of patient satisfaction, 100.58% ± 22.83% for improvements in work quality, and 104.07% ± 5.45% for improvements in economic performance. The improvement indices were 61.12% ± 13.2%, 60.47% ± 28.91%, 34.41% ± 22.96%, 49.22% ± 25.39% and 73.70% ± 5.24%, respectively.

**Table 2:** **Tangible achievements of QCC theme activities (%,****± SD)**

Theme activity	Reductions in internal errors	Reductions in costs	Increases in degree of patient satisfaction	Increases in work quality	Improvements in economic performance
Index					
Achievement index	109.8 ± 16.5	135.0 ± 50.3	126.3 ± 53.7	100.6 ± 22.8	104.1 ± 5.5
Improvement index	61.1 ± 13.2	60.5 ± 28.9	34.4 ± 23.0	49.2 ± 25.4	73.7 ± 5.2

### Intangible achievements

Evaluations of intangible achievements were made with respect to ability to solve problems, sense of responsibility, communication and coordination, self-confidence, team cohesiveness, initiative, approach to quality control, harmoniousness, language skills, sense of honor, and personal qualifications. Each aspect was assessed by self-evaluation by each QCC member, with the highest mark being 5 points and lowest 1 point. Activity Growth Value = mean mark after activity – mean mark before activity. The results show that after QCC activity, the medical workers showed improvement in ability to solve problems, sense of responsibility, communication and coordination, self-confidence, team cohesiveness, initiative, approach to quality control, harmoniousness, language skills, sense of honor, and personal qualifications. Five percent of QCC members showed an activity growth value of 0–1 points, 83% 1–2 points, and 12% above 2 points. The intangible achievements of QCC theme activities are shown in detail in Table 3.

**Table 3:** **Intangible achievements of QCC theme activities (****± SD)**

Theme activity	Reductions in internal errors	Reductions in costs	Increases in degree of patient satisfaction	Increases in work quality	Improvements in economic performance
Growth value					
Item evaluated					
Ability to solve problems	1.4 ± 0.6	0.9 ± 0.1	1.3 ± 0.6	1.5 ± 0.7	
Sense of responsibility	1.3 ± 0.8	0.9 ± 0.5	1.4 ± 0.8	1.2 ± 0.7	1.4 ± 0.9
Communication and coordination	1.2 ± 0.5	1.4 ± 0.5	1.4 ± 0.7	1.3 ± 0.9	
Confidence	1.5 ± 0.6	1.3 ± 0.6	1.3 ± 0.8	1.1 ± 0.8	1.2 ± 0.4
Cohesion	1.3 ± 0.6	1.1 ± 0.8	1.5 ± 0.7	1.3 ± 0.7	
Initiative	1.3 ± 0.7	1.1 ± 0.9	1.5 ± 0.6	1.5 ± 0.8	
Approach of QC	2.1 ± 0.9	2.8 ± 1.1	2.6 ± 0.9	2.1 ± 0.9	1.3 ± 0.3
Harmonious degree	1.3 ± 0.7	1.1 ± 0.4	1.1 ± 0.7	1.5 ± 0.8	1.0 ± 1.4
Language skills	2.0 ± 0.9				
Sense of honor	1.2 ± 0.5			1.3 ± 0.7	
Personal qualification	1.4 ± 0.8			1.3 ± 0.5	

*Growth values were self-marked by members of QCC; for each item the highest mark was 5 points, lowest 1 point.

## Discussion

The term tangible achievements here refers to achievements that can be quantified, such as defect rate, error rate,delay rate, number of complaints,or absentee rate. The quantities before and after the execution of measures meant to improve quality can be determined concretely. Usually, they can be represented in materialized format and their economic implications can be calculated. Examples include reductions in the number of internal errors in outpatient prescription dispensations, reductions in the density of baumanii infections in the ICU, decreases in the duration of equipment malfunction, increases in productivity, and decreases in error items in electronic nursing reports. Tangible achievements are more prone to attracting people’s attention and to become the focuses of organized achievement reports, publications, and rewards. After each goal value was set, in the theme activities, reductions in internal errors, reductions in costs, improvements in the degree of satisfaction, improvements in work quality, and improvements in economic performance, the achievement indices were 109.84% ± 16.47%, 135.04% ± 50.33%, 126.26% ± 53.69%, 100.58% ± 22.83%, and 104.07% ± 5.45%, respectively. The improvement indices were 61.12% ± 13.2%, 60.47% ± 28.91%, 34.41% ± 22.96%, 49.22% ± 25.39%, and 73.70% ± 5.24%, respectively. If the achievement index is too high (>150%), it indicates that the confidence level is low when the goal value is set. If the achievement index is too low (<80%), the improvability of the circle may have been overestimated.

The term intangible achievements refers to achievements that are hard to quantify, such as the personal growth of the circle head or members. Promoting QCC activity can stimulate work morale, improve the knowledge and skill of the staff, encourage active work attitudes, cultivate leadership, improve institutional image, reduce institutional costs, and increase degree of satisfaction. Intangible achievements can be represented with relative plots scored by the members themselves or by the chief director. The total number of evaluation items is best to even, in the range of 5–8. Balanced improvement is optimal. The index contrast values (e.g. growth value of theme activity) are calculated, and these values are positively related to the effectiveness of the results, i.e. the higher the value, the better the result. In the application and promotion of Zhejiang medical institution QCC to promote sustained improvements in quality, 5% of the QCC activities showed a growth value of 0–1 point, 83% 1–2 points and 12% above 2 points. This suggested that the medical workers had improve substantially in their ability to solve problems, sense of responsibility, communication and coordination, team cohesiveness, initiative, approach to quality control, harmoniousness, language skills, sense of honor, and personal qualifications. Improvements in approach to quality control were most prominent, with a growth value above 2 points.

QCC activity reflects the people-oriented core idea of management. It has been successfully carried out in overseas business circles for several decades and has been gradually extended to hospital management in recent years. The participants can learn about scientific quality management knowledge and tools. Training workers at the grassroots level can increase the awareness of amending problems and increase work efficiency. In this way, a harmonious work team can be built; the quality of medical service can be improved; and the hospital’s costs can be lowered. Through the bottom-up execution procedure of QCC, self-management of department personnel can be realized. This can also help administrative managers work more scientifically and rationally. Overall, medical quality can be broadly improved.

The standardization of QCC activity is to break down the current work procedure to every step and every actbased on the analysis and diagnosis of work flowchart. Then, according to scientific techniques, regulations, and practical experience, the work flowchart is improved with the goal of achieving better safety, quality, and economic performance. This promotes the formation of an optimal operating procedure. Standardization is the summarization of the practical experiences in QCC, an important step in improving QCC and one of the goals of QCC activity.
